# Political Trust and Sophistication: Taking Measurement Seriously

**DOI:** 10.1007/s11205-015-1182-4

**Published:** 2015-11-20

**Authors:** Sedef Turper, Kees Aarts

**Affiliations:** Department of Public Administration, University of Twente, P.O. Box 217, 7500 AE Enschede, The Netherlands

**Keywords:** Political trust, Political sophistication, Diploma democracy, The Netherlands, Measurement invariance

## Abstract

Political trust is an important indicator of political legitimacy. Hence, seemingly decreasing levels of political trust in Western democracies have stimulated a growing body of research on the causes and consequences of political trust. However, the neglect of potential measurement problems of political trust raises doubts about the findings of earlier studies. The current study revisits the measurement of political trust and re-examines the relationship between political trust and sophistication in the Netherlands by utilizing European Social Survey (ESS) data across five time points and four-wave panel data from the Panel Component of ESS. Our findings illustrate that high and low political sophistication groups display different levels of political trust even when measurement characteristics of political trust are taken into consideration. However, the relationship between political sophistication and political trust is weaker than it is often suggested by earlier research. Our findings also provide partial support for the argument that the gap between sophistication groups is widening over time. Furthermore, we demonstrate that, although the between-method differences between the latent means and the composite score means of political trust for high- and low sophistication groups are relatively minor, it is important to analyze the measurement characteristics of the political trust construct.

## Introduction

Political trust remains a prevalent topic in political science research. A vast amount of empirical research into consequences of political trust has shown that citizens with lower levels of trust in political institutions engage less often in institutionalized forms of political participation and more often undertake system-challenging political behavior (Hooghe and Marien 2013), and they are less likely to comply with the laws (Marien and Hooghe 2011). Having crucial implications for political participation and law-abiding behavior, political trust is considered to be an essential indicator of legitimacy in democratic regimes (Levi and Stoker 2000), and therefore seemingly declining levels of political trust in Western democracies over the last couple of decades have attracted considerable scholarly attention.

In this paper we address the question to which extent these trends, or the absence thereof, may be attributed to methodological artefacts, in particular shortcomings in the measurement of political trust. Cross-national and over-time studies attempting to chart and explain the trends in levels of political trust commonly employ additive indices of multiple survey items tapping on confidence in various political institutions. A major pitfall of these empirical studies is that they overlook basic measurement considerations of political trust. Firstly, the composite score model unrealistically assumes that trust in different political institutions equally weights and loads on the latent political trust construct. Secondly, the composite score model that is frequently employed in studies on political trust assumes indicators to be free from measurement errors. However, as Dekker (2012) illustrates, incidental measurements of political trust are highly sensitive to context. Measures from different simultaneous surveys and panel measures that are only 1 month apart from each other can still vary to a substantial degree. Measurement error and index artifacts should of course not be confused with real differences. Therefore, political trust measures need to be analyzed with statistical models that allow for different weights for each indicator and that control for random fluctuations in those measures.

The neglect of potential measurement problems of political trust raises doubts about the findings of earlier studies. Therefore, in the current study, we revisit the measurement of political trust through robust statistical analysis. We utilize European Social Survey (ESS) data for the Netherlands across five time points (2004, 2006, 2008, 2010, and 2012) and four-wave panel data from the *Panel Component of ESS* in the Netherlands. While panel data allows us to identify the measurement error, stable trait and over-time change components of political trust, the cross-sectional time series data from the ESS study allows us to explore the comparability of the political trust construct over a longer period of time.

We pay special attention to differences between *political sophistication* groups. Debates on the development of political trust have pointed to political sophistication as a key explanation (Bovens and Wille 2010; Dalton 2004). We start our analysis by testing measurement invariance of the political trust constructs among political sophistication groups in order to establish whether it is meaningful to compare the means of political trust constructs across groups and over time. We then utilize multi-group confirmatory factor analysis and autoregressive simplex model analysis to estimate and compare mean political trust scores for high and low political sophistication groups.

The empirical domain of this paper is restricted to the Netherlands, in the period 2004–2012. This restriction is substantively not important as the methods and—most likely—the results too can be generalized to other settings. First, the measurement items used in this study are common measures of political trust and are used in a variety of countries and settings. Secondly, although the Dutch case in the period 2004–2014 does exhibit some particularities with regard to the trend in political trust (see below), the structural relationships of political trust with other theoretical constructs are comparable to those in other countries (Catterberg and Moreno 2006; Hooghe and Marien 2013). Short-term deviations are attributed to idiosyncratic changes in political culture (Bovens and Wille 2008, pp. 301–303; van der Meer 2010).

The contribution of this paper to the literature is thus twofold. First, our analyses highlight the general importance of explicating and testing assumptions about the measurement properties of concepts. Taking such assumptions for granted may result in flawed conclusions about trends and relationships. Our findings show that composite score models that implicitly assume trust indicators to have equal validity and also to be free from measurement errors, tend to overestimate the level of trust for both high- and low-sophistication groups. Our analysis further illustrates that composite score models yield exaggerated estimates of the differences between the trust levels of high- and low-political sophistication groups when the condition of full scalar invariance is not met. Secondly, the current study revisits the relationship between political sophistication and political trust by employing statistically robust analyses. Our findings illustrate that, when compared to their less sophisticated counterparts, highly sophisticated citizens do display higher levels of trust in three key components of democratic regimes, namely; parliament, politicians, and political parties. However, in sharp contrast with earlier research these differences are found to be minor.

## Theoretical Background and Previous Research

### Trends in Political Trust

Political trust, generally defined as citizens’ confidence in political institutions, is an important indicator of political legitimacy—the belief in the righteousness of these political institutions and the regime of which they are part. A widespread belief in legitimacy is commonly regarded as a necessary condition for the survival of political regimes. Therefore, measuring the level and development of political trust may provide us with important information about the stability of political systems (Easton 1965, 1975). Trust in the political regime of a country constitutes a reservoir of good will for when the day-to-day performance of the regime fails to meet expectations. Given these crucial implications, political trust is often considered as an essential component of the civic culture that is necessary for stability of democratic systems (Almond and Verba 1963). Therefore, the seemingly decreasing levels of political trust in Western democracies over the last couple of decades (Dalton 2004, 2005; Klingemann 1999) have stimulated a growing body of research on the causes and consequences of political trust.

The presumed decline of political trust (and, by implication, the presumed decline of the legitimacy of political systems) is often attributed to long-term processes of modernization and globalization. At the level of the individual citizen, modernization implies among other things a rise in the level of education (Klingemann and Fuchs 1995). Modernization theory states that the increased level of education, in combination with increasing political interest and a decreasing respect for traditional authorities and institutions leads to a growing dissatisfaction of higher-educated citizens with the working of the political system (Aarts et al. 2014; Dalton 2004; Thomassen 2005). This growing dissatisfaction presumably translates into a decrease in the trust in political institutions, as these institutions apparently fail to do what modern citizens expect from them.

The effects of economic globalization, on the other hand, work in a different direction. Economic globalization primarily impacts on those citizens who in the process become less competitive on the labor market. In Western countries, these citizens are primarily workers in those production segments of the economy which can relatively easily be moved to other countries or other parts of the world where the costs of production can be optimized (see for example Kriesi et al. 2008). For these workers, replacement jobs are hard to find since their level of education is relatively low. Higher educated persons, in contrast, are much better suited for jobs in economic sectors that will survive the first waves of globalization, e.g. the service sector, research and development, and other jobs with relatively high qualifications. In short, economic globalization will likely have negative consequences for the lower strata of the labor market, which tend to be the lower-educated citizens. It is to be expected that these negative experiences will in turn lead to a decrease of political trust among this group.

Modernization and globalization are thus expected to have diverging effects on the development of political trust of different groups in society. The modernization process is assumed to lead to a decrease of political trust especially among the higher-educated citizens. This assumption is also known as “positive effects hypothesis”. The globalization process, on the other hand, is thought to lead to a decrease of trust among the lower-educated citizens. This is known as the “negative effects hypothesis” (Dalton 2005).

While the Netherlands has long been considered as an exceptional case with rising rather than declining levels of political trust, there has been a significant drop in political trust levels of Dutch citizens in the first half of the 2000s (Bovens and Wille 2008; Hendriks 2009). The sudden decline in the political trust levels stimulated a growing body of literature on political trust in the Netherlands (Bovens and Wille 2008; Hendriks 2009; van der Brug and van Praag 2007; van der Meer 2010).

Recent research into the case of the Netherlands has stressed the central role of education in understanding and explaining attitudes towards politics, including political trust. The phrase ‘diploma democracy’ has been coined by Bovens and Wille (2010). ‘Diploma democracy’ primarily refers to the alleged disappearance of lower-educated citizens from political life (Bovens and Wille 2012; Hakhverdian et al. 2011). Looking at political trust specifically, diploma democracy suggests that political sophistication acquired through education is the single strongest explanatory variable in understanding varying levels of political trust. According to this view, on top of the important consequences of economic globalization, the political arena itself has increasingly become a domain where only highly qualified, politically sophisticated citizens can exert influence and hence the less educated citizens become more and more alienated from politics (Bovens and Wille 2009, 2010). Supporters of the diploma democracy thesis argue that this feeling of exclusion from politics is causing cynicism and distrust among the less educated and less politically sophisticated citizens. These feelings of political exclusion add to the threat of economic exclusion as a result of economic globalization (Bovens and Wille 2010, pp. 415–416).

The empirical studies investigating the differences in political trust levels between the higher and lower educated citizens suggest that there is a gap between the two. The extent of this gap is not fully clear. Bovens and Wille (2010, pp. 412–413) quote several studies that find a positive relationship between education and trust. Other studies point to rather weak relationships, especially when other explanatory factors are taken into account (e.g. Listhaug and Wiberg 1995). However, the neglect of potential measurement problems of political trust raises doubts about the findings of these earlier studies documenting the gap between the levels of political trust among high and low sophistication groups.

### Political Trust: Critical Look at the Common Analytical Strategies

In the current section, we discuss the common analytical strategies in political trust research and their methodological shortcomings that we briefly presented already in the introduction section. In the study of political trust, two practices seem to be widespread. The first of these is single-item measurement. Individual items asking about trust in parliament, or trust in the government, are analyzed as if these are valid and reliable indicators of the concept to be measured (Hetherington and Rudolph 2008; Newton 2001; Rudolph and Evans 2005; van der Meer 2010). However, when a concept is measured by a single item, the validity and reliability of the measure cannot be assessed at all, as it is impossible to distinguish between the various components of the measurement (true score, systematic error, random error) through single occasion measurement designs.^1^ Single item measurement thus results in a poor form of operationalization. Not only is “trust” defined as what is measured by a survey item, but the survey item also deliberately refers to only one object of political trust.

The second, and more commonly employed practice is that researchers construct political trust indices by adding or averaging the levels of confidence that individuals have for a set of political institutions (Bovens and Wille 2008; Brewer et al. 2005; Catterberg and Moreno 2006; Hendriks 2009; Marien and Hooghe 2011; van der Brug and van Praag 2007). However, these sum or composite score models that are frequently employed in political trust research have often been criticized for making a couple of unrealistic assumptions that jeopardize the robustness of statistical findings (Saris and Gallhofer 2007, pp. 314–315). Firstly, in sum or composite score models, each indicator is assumed to be equally contributing to the underlying latent construct. As far as the political trust constructs are concerned, utilizing these models means that the measures of trust in different political institutions equally weight and load on the latent political trust construct. However, previous research illustrates that validity coefficients for individual trust indicators vary to a substantial extent, and therefore the regression coefficients for each indicator needs to be corrected for by applying appropriate weights while calculating sum scores for obtaining robust findings (Saris and Gallhofer 2007). Secondly, composite score models further assume constitutive measures of the construct (the survey items) to be free of measurement errors. In other words, in these models, indicators are treated as perfectly reliable measures of the underlying political trust construct. Yet, MTMM experiments^2^ conducted with ESS measures tapping on trust in institutions demonstrate that political trust indicators are susceptible to measurement errors.^3^ Furthermore, not taking measurement errors into account also means that the reliability of survey questions is assumed to be equal across groups. Previous research, however, demonstrated that the reliability of the survey items is significantly contingent upon respondents’ levels of political interest and education (Alwin and Krosnick 1991; Judd et al. 1981; Judd and Milburn 1980), and hence measurement errors are not invariant for low and high political sophistication groups. Therefore, when comparing political trust scores across sophistication groups the differences in reliability of trust indicators need to be factored in the statistical models.

Confirmatory factor analysis (CFA) models can overcome the shortcomings of the analytical approaches mentioned above by allowing different factor loadings and accounting for measurement errors. CFA models are frequently employed in the study of various attitudes such as attitudes towards immigration (Meuleman et al. 2009; Savelkoul et al. 2012), welfare (van Oorschot and Meuleman 2012) and democracy (Ariely and Davidov 2010) with the purpose of comparing latent constructs means across populations and over-time. However, there are only a few studies on political trust that actually employ these measurement models that takes validity and reliability considerations into account (Allum et al. 2011; André 2014; Davidov and Coromina 2013; Poznyak et al. 2014; van Elsas 2014).

## Current Study

In the current study, we revisit the ‘diploma democracy’ argument that was put forward by several scholars attempting to chart and explain recent trends in levels of political trust in the Netherlands. Scholars endorsing the ‘diploma democracy’ argument stress that politically less sophisticated citizens become increasingly alienated from politics through the global processes that the negative effects hypothesis postulates, and that they display substantially lower levels of political trust when compared to their politically sophisticated counterparts. However, these studies often lack robust statistical methods to control and account for measurement errors and also for possibly different latent factor models. Therefore, we start our analysis with testing across-group and over-time measurement equivalence of the political trust construct among high and low political sophistication groups. Then we run multi-group confirmatory factor analyses and auto-regressive simplex models to obtain and compare mean political trust scores of these two political sophistication groups.

### Data

For the first part of the study, we utilize European Social Survey data from 2004 to 2012 for the Netherlands. The data used for the second part of the study is obtained from *Panel Component of European Social Survey*, a developmental project aiming at facilitating biannual cross-sectional ESS survey and funded by the Netherlands Organization for Scientific Research (NWO). The panel study started with ESS Round-5 in October 2010 with the participation of 1829 respondents recruited through probability sampling of households and representative of the Dutch population over the age 16. After completing the ESS survey, all respondents were asked to take part in the panel study and 1501 of those respondents agreed to take part in the panel study.^4^ The subsequent waves of the study were conducted between May 2011 and January 2013 with 8 month intervals in-between. The last wave of the panel study completed with the participation of 647 respondents. The response rates for the panel study were 0.60 for the initial ESS study and 0.72, 0.70 and 0.86 for the subsequent waves, respectively.

For the current study, we used list-wise deletion, which yielded total samples of 1831, 1841, 1745, 1785, 1812 respondents for five consecutive waves of ESS (2004, 2006, 2008, 2010, 2012), respectively, and a total sample of 543 respondents in the panel study. While the mean age was 49.2, 48.6, 49.0, 50.0, and 51.1, the average years of formal schooling is recorded as 12.3, 13.3, 13.3, 13.4, and 13.6 in the final samples of subsequent waves of ESS, respectively. In the panel study, the final sample had a highly even gender distribution of 273 male (50.3 %) and 270 female (49.7 %) respondents, with a mean age of 51.6 (SD = 16.24) and with an average of 13.9 (SD = 4.07) years of formal schooling.

### Variables

We measure political sophistication in two categories (1 = high; 2 = low). We assess political sophistication on the basis of educational attainment and level of interest in politics^5^ (Table 1). Although there is no causal relation, there is a strong correlation between education, political interest and political sophistication (Althaus 2003; Highton 2009; Luskin 1990). The high political sophistication group consists of those respondents holding a university or university of applied sciences degree, who also expressed interest in politics (very interested or quite interested), at the time of the first interview. By the same token, the low political sophistication group is composed of those respondents without a college or vocational degree and/or those expressed little or no interest in politics.

**Table 1:** Political sophistication

Education	Political interest	
	Not interested at all/A little interested	Interested/Very interested
No university degree	*Low*	*Low*
University degree	*Low*	*High*

Cells denoted by ‘*Low*’ and ‘*High*’ refers to ‘low’ and ‘high’ political sophistication groups, respectively

Consequently, in the consecutive waves of the ESS study, approximately 30 % of the respondents are grouped into the high political sophistication group (28.9 % in 2004; 30.6 % in 2006; 33.4 % in 2008; 30.2 % in 2010; 29.6 % in 2012). Similarly, in the panel study, the high political sophistication group consisted of 193 respondents (35.5 %), while 350 of those respondents are categorized into the low political sophistication group (64.5 %).^6^ As Table 2 illustrates, high and low political sophistication groups display different demographic characteristics. The high political sophistication groups contain a significantly higher proportion of males when compared to the low political sophistication groups. Age and average years of formal schooling differences between high and low political sophistication groups were also found to be statistically significant in all datasets we employed in the current study. The mean age of the low political sophistication groups was on average 5 years higher when compared to their high sophistication group counterparts, whereas average years of formal schooling was observed to be higher among the high sophistication groups.

**Table 2:** Demographic characteristics of political sophistication groups

	Sample size (raw numbers)		Gender (perc. of males)		Age (mean, SD)		Years of formal schooling (mean, SD)	
	High	Low	High	Low	High	Low	High	Low
ESS 2004	530	1301	50.9	38.4	45.4 (16.0)	50.7 (17.6)	15.5 (3.3)	11.0 (3.2)
ESS 2006	563	1277	50.8	44.6	45.2 (16.4)	50.0 (17.9)	16.3 (4.4)	11.9 (3.9)
ESS 2008	582	1163	51.7	43.4	45.4 (16.9)	50.7 (17.8)	16.3 (4.0)	11.8 (3.7)
ESS 2010	539	1246	54.2	42.5	46.3 (16.3)	51.6 (17.6)	16.8 (3.9)	11.9 (3.5)
ESS 2012	537	1275	51.6	44.5	46.9 (17.1)	52.8 (18.0)	17.2 (3.7)	12.1 (3.5)
ESS Panel	193	350	58.3	46.0	48.1 (16.5)	53.5 (15.8)	16.8 (3.9)	12.3 (3.2)

Labels ‘Low’ and ‘High’ refers to ‘low’ and ‘high’ political sophistication groups, respectively

We further test the criterion validity of our political sophistication variable through inspection of its relation to political participation. Politically sophisticated citizens are expected to display higher levels of political involvement compared to their less sophisticated counterparts. In line with the expectations, we observe different levels of political involvement among high and low political sophistication groups. In all datasets, high political sophistication groups were found to display higher levels of engagement in conventional and unconventional political participation compared to their less sophisticated counterparts; they have a higher turn-out rate in the last elections, they more often sign petitions and work in organizations and associations.

To operationalize political trust we follow Dalton’s definition of key elements of representative democracy (Dalton 2004, p. 37), and we measure political trust by expressed levels of trust in three representative institutions, namely; national parliament, politicians, and political parties (0 = no trust at all; 10 = complete trust). We expect these three indicators to load on a single factor as earlier theoretical and empirical research illustrated that trust in representative institutions forms a one-dimensional construct^7^ (Hooghe and Marien 2013; Marien and Hooghe 2011; Newton and Zmerli 2011; Quintelier and Hooghe 2011; Zmerli 2006). Furthermore, we expect that political trust measured in the form of confidence in the political institutions displays a one-dimensional structure regardless of respondents’ level of political sophistication (Hooghe 2011).

### Analytical Models

#### Measurement Model and Measurement Invariance Test

The comparison of means of multiple-item constructs across groups and/or across time is problematic unless the measurement invariance of these constructs can be established through statistical testing (Beuckelaer and Swinnen 2011; Davidov and Coromina 2013; Steenkamp and Baumgartner 1998; Van Deth 2009). While various statistical techniques have been developed to test for measurement equivalence such as item response theory and latent class analysis, the most frequently used approach to test measurement equivalence is the multi-group confirmatory factor analysis model (Jöreskog 1971; Bollen 1989; Steenkamp and Baumgartner 1998; for a review of statistical tests for detecting measurement invariance, see also Braun and Johnson 2010).

**Fig. 1:**
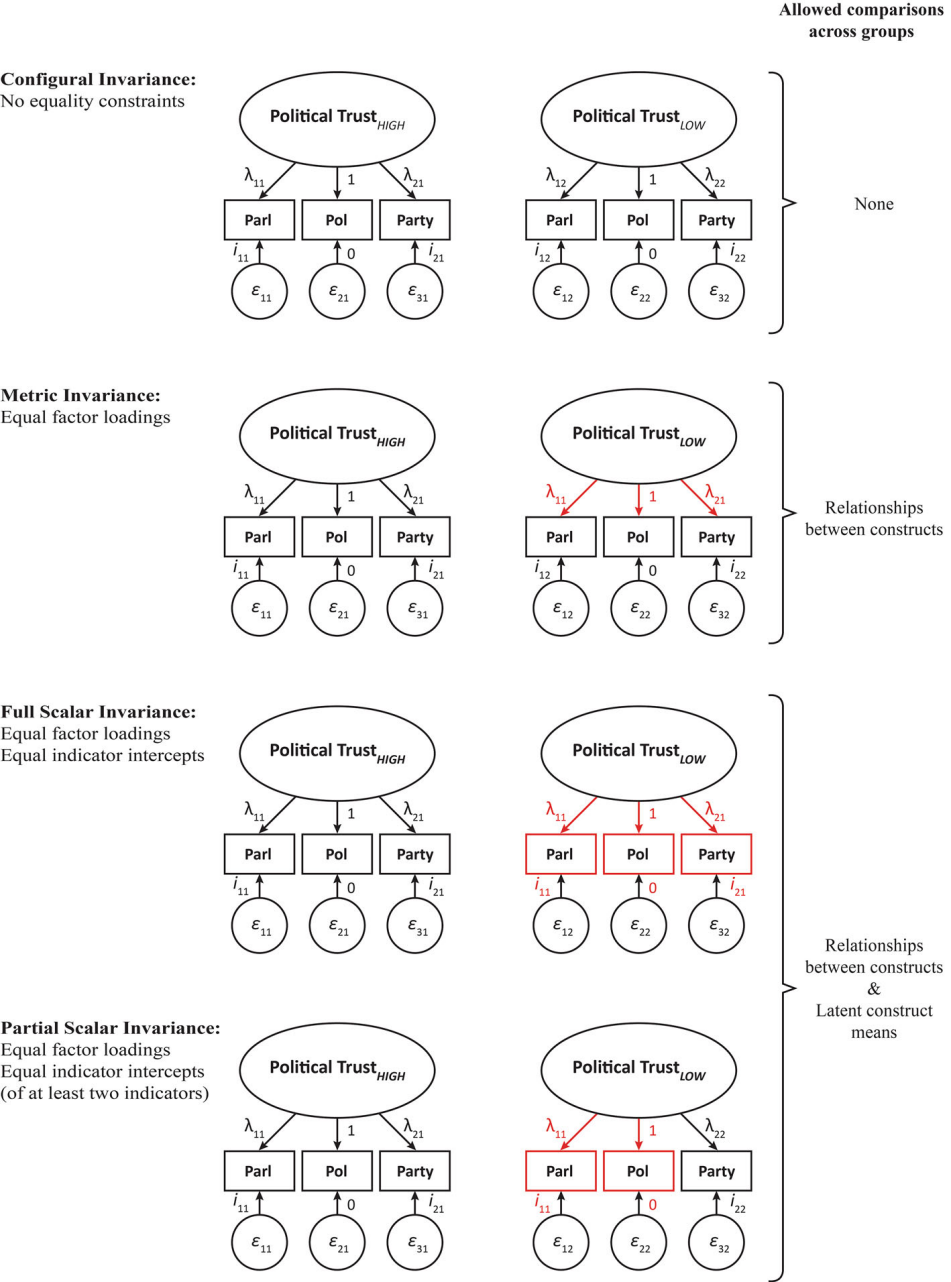
Measurement invariance models

To establish the extent of measurement invariance through MGCFA models three hierarchical levels of measurement invariance need to be tested, namely; configural invariance, metric invariance and scalar invariance (Fig. 1). These models are nested, and the test for invariance is conducted by comparing global model fit indices. Configural invariance refers to the model where only the factorial structures are invariant across groups whereas all the parameters for the model are freely estimated in each group. Configural invariance is the least restrictive level of invariance and it constitutes the baseline model for more restrictive tests.

Metric invariance requires factor loadings of indicator variables to be equivalent across groups. If this restriction, applied to the configural model does not significantly deteriorate the overall model fit, then metric invariance holds, and the relationships between latent constructs and other constructs can be meaningfully interpreted across groups. Metric invariance constitutes a more restrictive level of invariance when compared to configural invariance, and it allows for comparisons of regression coefficients across groups. However, a meaningful comparison of latent construct means requires an even more restrictive equivalence, namely scalar invariance. In the scalar invariance model, not only the factor loadings of indicators are restricted to equality but also all the indicator intercepts are required to be invariant across groups. This is called full scalar invariance. When full scalar invariance does not hold, latent means can still be compared if partial scalar invariance can be attained. Partial scalar invariance requires factor loadings and indicator intercepts of at least two indicators of each latent construct to be invariant across groups.

For evaluating the model fit of each invariance model we employ several goodness-of-fit indices, namely; root mean square error of approximation (RMSEA), standardized root mean square residual (SRMR), comparative fit index (CFI), and Tucker-Lewis index (TLI). We consider RMSEA values lower than 0.05 and SRMR values lower than 0.09, together with CFI and TLI values higher than 0.90 as an indication of acceptable fit for the models. In order to evaluate whether the restrictions introduced at each level of invariance test decrease the model fit within acceptable limits, we adopt a bottom-up strategy where we start with the least restrictive model and proceed with more restrictive models. We use Chi square (χ^2^) test statistics and CFI values for evaluating the decrease in the model fit as we move from the configural to metric and scalar invariance models. We consider Chi square test *p*-values greater than 0.05, and a change in CFI values less than or equal to 0.01 as an indication that the null hypothesis of invariance should not be rejected (Cheung and Rensvold 2002). Furthermore, we examine the modification indices (MI) and expected parameter change (EPC) for detecting possible model misspecifications.

#### Multi-Group Latent Mean Comparison

**Fig. 2:**
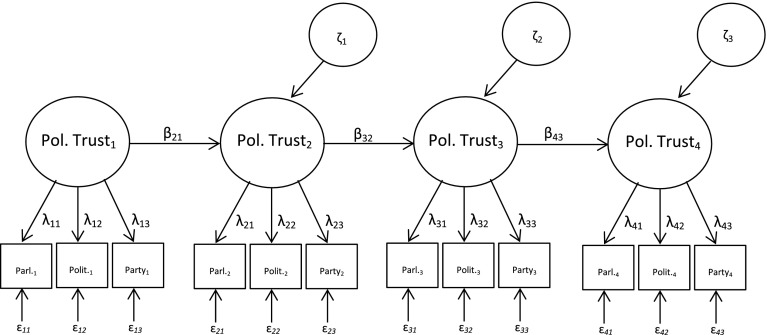
Auto-regressive (simplex) model with four waves

For estimating the mean levels of political trust among high and low political sophistication groups across five time periods (2004, 2006, 2008, 2010, 2012) with ESS data, we utilize single factor MGCFA model. In the panel study, on the other hand, for identifying the mean structure of the latent political trust construct over time for high and low sophistication groups we utilize an autoregressive (simplex) model. Figure 2 illustrates the path diagram of an autoregressive model for a four-wave panel design. In the figure, the observed variables are labelled as *Parl*_*i*_ *, Polit*_*i*_ *, Party*_*i*_ and they represent trust in parliament, trust in politicians and trust in political parties at *i*th wave of the panel study, respectively. In the model, *Parl*_*i*_ *, Polit*_*i*_ *, Party*_*i*_ are modelled to be indicators of latent political trust construct at time *i*. The parameters denoted by λ_ij_ and ɛ_ij_ refer, respectively, to the factor loading and error term of the *j*th indicator at time *i*. To put it differently, while lambda parameters indicate the degree to which each trust indicator represents the underlying political trust construct, error terms illustrate the extent to which the observed variables are affected by random measurement errors.

The model assumes a lag-1 or Markovian process, meaning that the distribution of political trust construct at time *t* is dependent only on its distribution at time *t* − 1, and not directly dependent on the earlier distributions. The parameters β21, β32 and β43 can be interpreted as stability coefficients of the political trust construct over time. The variances of political trust construct are denoted by ζ1, ζ2, and ζ3, and they represent the extent that political trust changes over time.

For the current study, we preferred the marker variable method (Little et al. 2006) for estimating latent political trust means for low and high sophistication groups. The latent mean estimates obtained through the marker-variable method preserve the metric of the marker indicator, and hence provide us with a latent mean estimate on the same scale as the marker variable. Since we utilize marker political trust indicators measured on an 11-point scale, the estimates for latent political trust means will also be adopting the same scale.

### Results

We start our analysis by testing the measurement equivalence of political trust construct across high and low political sophistication groups between the years 2004 and 2012 by utilizing five rounds of the ESS dataset for the Netherlands. As we mentioned earlier, we employ MGCFA analysis starting with the least restrictive invariance model and gradually moving towards the more strict invariance models. Table 3 summarizes the global fit indices for MGCFA models for different levels of measurement invariance. We do not provide global fit statistics for the configural invariance in the table below, as the model for a single latent construct with three indicators is just identified and fit indices cannot be obtained.

**Table 3:** Global fit measures for models testing for measurement invariance of political trust

	χ^2^	*df*	CFI	TLI	RMSEA	SRMR
Metric invariance	28.047	18	0.998	0.997	0.025	0.029
Full scalar invariance	231.896	36	0.968	0.973	0.078	0.057
Partial scalar invariance	69.896	30	0.993	0.993	0.038	0.037

χ^2^ = Chi square, *df* degrees of freedom, *CFI* comparative fit index, *TLI* Tucker-Lewis index, *RMSEA* root mean square error of approximation, *SRMR* standardized root mean

The global model fit indices for the metric invariance model reported in the first row of Table 3 indicates a good fit of the model with RMSEA and SRMR values below 0.05, as well as, CFI and TLI values above 0.90. Inspection of MI and EPC statistics also did not reveal any significant model misspecifications. Based on the model results, we conclude that factor loadings of the political trust indicators are equivalent across high and low political sophistication groups at five time points. Next, we proceed with testing scalar measurement invariance. The model fit indices for scalar invariance model indicate that introducing equality constraints on indicator intercepts resulted in a significant reduction in model fit. As we moved from the metric invariance model to the scalar invariance model, the increase in Chi square was highly significant (Δχ^2^(18) = 203.849, *p* < .000). In a similar vein, CFI and TLI values also decreased more than .01 points and the RMSEA value for the invariance model fell below the acceptable limit of 0.05. Thus we reject the full scalar invariance model. In order to assess partial scalar invariance, we released the equality constraints on six indicator intercepts through inspection of MI and EPC values for model misspecifications. Although the increase in Chi square relative to the metric invariance model was still significant (Δχ^2^(12) = 41.849, *p* < .000), RMSEA values improved significantly and the decrease in CFI fell within acceptable limits for the partial scalar invariance model. Furthermore, MI and EPC values did not suggest any considerable model misspecifications. Therefore, partial scalar invariance for the high and low political sophistication groups across five time points between 2004 and 2012 is supported. This means that latent political trust means can be meaningfully compared across high and low political sophistication groups at each measurement point.

Having established partial scalar invariance, we proceed with estimating the latent political trust means for high and low political sophistication groups. Table 4 presents political trust mean estimates from the CFA models for each political sophistication group at each measurement point. As mentioned earlier, the latent political trust means estimated by the model adopts the response scale of the indicator variables, and hence they should be interpreted as mean scores of a latent scale ranging from 0 to 10.

**Table 4:** Implied latent means for political trust, 2004–2012

	2004	2006	2008	2010	2012
High sophistication	5.213	5.457	5.645	5.775	5.737
Low sophistication	4.411	4.861	4.975	4.935	4.704
Difference in group means	0.802	0.596	0.670	0.840	1.033

Estimates are based on CFA models with marker indicator method

Comparison of latent mean estimates for high and low political sophistication groups at each time point reveals that politically more sophisticated citizens consistently display higher levels of political trust than their less sophisticated counterparts at each of the five measurement points (Table 4). More specifically, the model implies that political trust means for high political sophistication groups range between 5.21 and 5.77, whereas latent mean estimates for the low political sophistication group fluctuate between 4.41 and 4.98 levels. However, closer inspection of the latent mean differences between high and low political sophistication groups reveals that differences are indeed minor. Comparison of high and low political sophistication groups on mean trust levels between 2004 and 2012 shows that the differences between group means are on average 0.79 points, and the gap between political trust levels of high and low political sophistication groups never exceeds one point on an eleven point scale.^8^

Furthermore, we compare latent and composite score estimates for the two sophistication groups across five consecutive waves of the ESS. Table 5 reports the mean estimates obtained through the composite score models for the identical groups and time points utilized for estimating latent political trust means. The comparison of composite score means with previously presented latent means (Table 4), suggests that the composite score models yield slightly different results. Composite score models tend to overestimate political trust means in both high and low political sophistication groups, yet to differing extents. While the composite score models estimated political trust means on average 0.05 points higher than CFA models for the low sophistication groups, the overestimation bias was 0.12 points for the high sophistication groups. Consequently, the differences between high and low political sophistication groups tend to be greater when political trust means are obtained by utilizing composite score models.

**Table 5:** Composite score means for political trust, 2004–2012

	2004	2006	2008	2010	2012
High sophistication	5.253	5.622	5.805	5.886	5.867
Low sophistication	4.431	4.938	5.015	4.991	4.781
Difference in group means	0.822	0.684	0.790	0.895	1.086

Estimates are based on composite score models

In the second part of this study, we investigate the mean trust levels among high and low political sophistication groups by utilizing panel data with four waves. As discussed earlier, comparison of latent construct means can only be meaningful if the structure of the latent factor is similar in each group and at each time point of measurement. Therefore, we again start our analysis by testing the measurement equivalence of political trust constructs among high and low sophistication groups across four waves of measurement. To this end, we conduct a multi-group confirmatory factor analysis with eight groups to test for across sophistication groups and for over-time invariance of political trust construct.

Table 6 summarizes measurement invariance test results with four wave panel data.^9^ The model fit indices for the metric invariance model are high, with an RMSEA value of 0.000, as well as CFI and TLI values above 0.99. Hence, metric invariance of the political trust construct is supported. In a similar vein, the full scalar invariance model fits the data reasonably well with an RMSEA value below 0.05 and SRMR value below 0.09. Although the Chi square increase in comparison with the metric invariance model is significant (Δχ^2^(14) = 28.782, *p* < 0.000), the increase in CFI value (ΔCFI = 0.004) is well below the cut-off criteria of 0.01. Further inspection of MI and EPC statistics also does not suggest any considerable model misspecifications, and hence the full scalar invariance model was supported in the panel study.

**Table 6:** Global fit measures for models testing for measurement invariance of political trust

	χ^2^	*df*	CFI	TLI	RMSEA	SRMR
Metric invariance	7.704	14	1	1	0.000	0.038
Full scalar invariance	36.486	28	0.996	0.997	0.033	0.052

χ^2^ = Chi square, *df* degrees of freedom, *CFI* comparative fit index, *TLI* Tucker-Lewis index, *RMSEA* root mean square error of approximation, *SRMR* standardized root mean

Our findings of the measurement invariance test allow meaningful comparisons of political trust means and regression coefficients between the two sophistication groups across four waves of panel study. Hence, we proceed with examination of the latent political trust means through a two group auto-regressive (simplex) model. Table 7 presents the latent means for political trust among high and low sophistication groups obtained through the auto-regressive model (denoted by κ) as well as means computed through the composite score model (denoted by μ). Inspection of the mean estimates again reveals that politically more sophisticated citizens display slightly higher levels of political trust when compared to less sophisticated citizens. The gap between trust levels of the two sophistication groups is however only minor. The panel study shows that, on average, politically more sophisticated citizens display 0.7 points higher levels of trust on a political trust latent trait measured on an 11-point scale, which amounts to a difference of 7 % points.^10^

**Table 7:** Implied latent means and composite score means for political trust

	*Wave 1*		*Wave 2*		*Wave 3*		*Wave 4*	
	*κ*	μ	*Κ*	μ	*κ*	μ	*Κ*	μ
High sophistication	5.789	5.874	5.834	5.995	5.681	5.819	5.729	5.872
Low sophistication	5.112	5.211	5.311	5.474	5.090	5.220	4.777	4.962
Difference in group means	0.677	0.663	0.523	0.521	0.591	0.599	0.952	0.910

‘κ’ denotes latent mean estimates, ‘μ’ denotes composite score means

Comparison of the latent and composite score mean estimates illustrates that the composite score model yields slightly higher levels of political trust in both sophistication groups at each measurement point. However, in the panel study where full scalar invariance is established the overestimation bias applies to high and low sophistication groups alike. In other words, when full scalar invariance holds across groups and over-time, the differences between mean political trust levels among high and low sophistication groups are observed not to be different than those estimated through composite score model. In the previous analysis of five rounds of ESS data where only partial scalar invariance was present, the bias was found to be different for the high- and low-sophistication groups, and hence the differences between sophistication groups were slightly exaggerated when composite score model was used. This in turn suggests that composite score model yields similar results as the latent means model only when the full scalar invariance condition has met.

## Conclusion and Discussion

In the current study, we have investigated the measurement properties of political trust construct composed of three political trust items by utilizing two datasets. First, we compared five successive rounds of the European Social Survey in the Netherlands (2004–2012), and secondly we analyzed political trust in a four-wave panel survey in the Netherlands (2010–2012). We determined the extent to which measurement invariance is applicable to political trust. The analysis of political trust in the panel study pointed to full scalar invariance, which means that in this case a composite score model would basically be equivalent to a factor score model. In the panel study, although we do find minor differences in the mean estimates of political trust for high- and low-sophistication groups produced by composite and factor score models, these two models provide similar estimates of the differences between the levels of political trust for high- and low-sophisticated. In the cross-sectional ESS studies covering biannual measures of political trust indicators over an 8 years’ time span, on the other hand, the analysis pointed to partial scalar measurement invariance, suggesting that the level of measurement errors involved in measures were not invariant across time and across political sophistication groups. Comparing latent means with the results of a sum score model, we found that the sum score model tends to result in slightly exaggerated differences in trust between different sophistication groups. Our findings illustrate that commonly employed composite score models produce unbiased estimates of the differences between the levels of political trust for high- and low political sophistication provided that the full scalar invariance condition has been met.

The current study provides much needed evidence for whether the observed differences in political trust levels of high and low sophistication groups are genuine, or merely methodological artefacts stemming from ignoring measurement properties of political trust construct. Confirming the findings of previous research, the current study illustrates that the highly sophisticated citizens display higher levels of political trust when compared to their less sophisticated counterparts. However, in a sharp contrast to earlier research,^11^ the findings of the current study illustrate that the differences between political trust levels of high and low sophistication groups are minor. Comparing the means of the latent political trust construct, the differences between high- and low-sophistication groups appeared to be relatively small in the Netherlands. Furthermore, we detected traces of a widening of the gap between these two groups starting from 2006. Our analysis with five successive rounds of European Social Survey in the Netherlands demonstrates that the gap between the trust levels of high- and low sophistication groups steadily increases between 2006 and 2012. A possible explanation for this seemingly growing gap is that the start of the time series occurred at a highly atypical level of trust. In 2004, shortly after the rise of the populist List Pim Fortuyn, and the murders of Fortuyn in 2002 and of Theo van Gogh in 2004, trust in political institutions in the Netherlands was temporarily rather low across the board.

Our research findings point at important implications for the practitioners of the political trust research. Although, cross-sectional time series data provide researchers with valuable information for the study of trends in political trust, findings of the current study demonstrate that repeated measures of identical political trust items cannot ensure comparability of political trust construct over time and across political sophistication groups. Our research findings suggest that overlooking the measurement properties of political trust constructs can lead to biased estimates. Therefore, we underline that researchers should pay attention to measurement invariance, especially when dealing with longitudinal data covering larger time intervals. Further on this point, our findings illustrate that comparing measures of political trust obtained with identical survey items over a longer period of time may be misleading when composite score models are utilized in the analysis. Therefore, we strongly recommend practitioners of political trust research to employ measurement invariance tests and to utilize composite score models for political trust, if and only if the full scalar invariance condition has been met.

Our findings further illustrate that political trust is a similar construct and has an equivalent meaning across high and low political sophistication groups in the Netherlands for the time period covered in the current study. This implies that researchers can make meaningful comparisons of political trust levels and regression coefficients between political trust and other theoretically related constructs across political sophistication groups by adopting structural equation model approach.

However, some limitations of this study need to be acknowledged, Firstly, the current study exclusively focuses on political trust in representative institutions, and in so doing, overlooks two other dimensions of political trust, namely trust in regulatory institutions (Denters et al. 2007) and trust in international or supranational political institutions (Schnaudt 2013; Van Deth 2000). Future research should try to validate our findings regarding the differences in mean levels of political trust between high and low political sophistication groups, and investigate whether these differences also apply to other dimensions of political trust. Secondly, it is important to note that the multi-group confirmatory factor analysis applied in the current study is subject to criticism. As Steenbergen asserts, multi-group confirmatory factor analysis tends to over fit in small samples by extracting too many factors, and hence requires relatively large sample sizes (Steenbergen 2000). Additionally, there is no consensus over the cut-off criteria for the goodness of fit indices, and Saris et al. (2009) offer alternative procedures to evaluate structural equation models instead of those utilized in the current study.

Furthermore, our research findings concerning the growing gap between the political trust levels of low-and high sophistication groups should be interpreted with caution. Although our findings point to a widening of the gap between the political trust levels of highly and less sophisticated individuals from 2006 onwards, the dataset we utilized for the study covers a relatively short period of time and hence falls short of providing us with compelling evidence for a trend analysis. Therefore, future research should try to validate these research findings by utilizing measures of political trust obtained over a longer period of time. Our study establishes some specific directions for future research on the relationship between political trust and political sophistication. Findings of the current study clearly establish that the differences in mean levels of political trust between highly and less sophisticated individuals are not merely methodological artifacts but are robust. Building on this point, future research should focus on the causal mechanisms that bring about the differences in political trust levels across political sophistication groups. We invite more research covering a longer period of time and a variety of political settings, which will make it possible to employ more sophisticated multivariate analyses for disentangling the causal mechanisms that can account for differing levels of political trust among high- and low- political sophistication groups. Additionally, findings of the current study demonstrate that overlooking the measurement properties of political trust can lead to biased results when levels and relationships of political trust constructs are compared across different (sub-)populations. Therefore, future research should also revisit and try to validate the findings of the earlier studies making comparisons across different samples of the respondents by employing measurement invariance tests and structural equation modeling approach.

To conclude, the current study illustrates that high- and low political sophistication groups display different levels of political trust even when measurement characteristics of political trust are taken into consideration. However, the relationship between political sophistication and political trust is weaker than it is often suggested by earlier research. Our findings also provide partial support for the argument that the gap between sophistication groups is widening over time. Furthermore, this study demonstrates that, although the between-method differences between the latent means and the composite score means of political trust for high- and low-sophistication groups are relatively minor, it is important to *analyze* the measurement characteristics of the political trust construct rather than simply *assume* that these are good enough. Precisely because political trust is an important concept it needs to be measured as accurately as possible. Trends and relationships are easily overestimated when the measurement is faulty.

## Footnotes

1 Reliability of single items can be assessed under the (random) parallel test model assumptions through item response theory (IRT) and generalizability theory.

2 MTMM stands for Multi-Trait Multi-Method; see (Campbell and Fiske 1959).

3 The MTMM experiments conducted in the ESS Round 2 estimated the reliability of trust in parliament, legal system and politicians to be 0.82, 0.85 and 0.84, respectively (for further information on these MTMM experiments see http://sqp.upf.edu/).

4 A random selection of 500 panel participants are asked for Computer Assisted Personal Interviewing (CAPI), and the remainder for Computer Assisted Web-based Interviews (CAWI).

5 Although education is used as a proxy for political sophistication in a vast majority of studies on political trust, an operationalization on the basis of levels of education and political interest is preferred as it better reflects the inherent meaning of the concept.

6 The overall panel attrition rate is observed to be higher for the low political sophistication group (0.67) than for the high sophistication group (0.56). Comparison of initial and final samples in terms of key demographic variables suggests that missing are at random for both low and high sophistication groups. However, we observed that those less sophisticated respondents who stayed in the panel and participated in all four waves were slightly more involved in politics than those who opted out.

7 Even in those empirical studies where two or more dimensions of political trust are detected, confidence in representative institutions found to be loading on one single factor. Therefore we expect that confidence in these three institutions to form a unified construct (Schnaudt 2013).

8 Replication of the analysis with low- and high-education groups (Low Education = no university or university of applied sciences degree, High Education = university or university of applied sciences degree) yielded to similar results.

9 As before we do not provide global fit statistics for configural invariance as the model for a single latent construct with three indicators is just identified and fit indices cannot be obtained.

10 Replication of the analysis with low and high educational attainment groups (Low Education = no university or university of applied sciences degree, High Education = university or university of applied sciences degree) yielded to similar results.

11 For example, Bovens and Wille cite a study by Dekker and van der Meer (in Dekker et al. 2009) to illustrate that educational groups differ significantly in their levels of political trust. They suggest that 57 % of the less-educated respondents are dissatisfied with politics as opposed to 34 % of the better-educated group. However, these findings partly stem from another common practice employed in the study of political trust, namely collapsing of the measurement scale of political trust scores into two categories, low and high. Dekker’s analysis already illustrates that the differences between educational groups are as small as .8 points on a 10-point scale. Yet, 5.5 is used as the cut-off point to sort predicted probabilities from a logistic regression. Although the regression coefficients are small, since the constant is around the cutoff point, most of the lower-educated people are predicted to have scores below the cut-off point. This is largely an artifact of collapsing categories of the trust scale.

## References

[CR1] Aarts K, Thomassen JJ, van Ham C, Thomassen JJ (2014). Globalization, representation, and attitudes towards democracy. Elections and representative democracy: Representation and accountability.

[CR2] Allum N, Read S, Sturgis P, Davidov E, Schmidt P, Billiet J (2011). Evaluating change in social and political trust in Europe using multiple group confirmatory factor analysis with structured means. Cross-cultural analysis. Methods and applications.

[CR3] Almond GA, Verba S (1963). The civic culture: Political attitudes and democracy in five nations.

[CR4] Althaus SL (2003). Collective preferences in democratic politics: Opinion surveys and the will of the people.

[CR5] Alwin DF, Krosnick JA (1991). The reliability of survey attitude measurement the influence of question and respondent attributes. Sociological Methods & Research.

[CR6] André S (2014). Does trust mean the same for migrants and natives? Testing measurement models of political prust with multi-group confirmatory factor analysis. Social Indicators Research.

[CR7] Ariely G, Davidov E (2010). Can we rate public support for democracy in a comparable way? Cross-national equivalence of democratic attitudes in the World Value Survey. Social Indicators Research.

[CR8] Beuckelaer A De, Swinnen G, Davidov E, Schmidt P, Billiet J (2011). Biased latent variable mean comparisons due to measurement noninvariance: A simulation study. Cross-cultural analysis: Methods and applications.

[CR62] Bollen KA (1989). Structural equations with latent variables.

[CR9] Bovens M, Wille A (2008). Deciphering the Dutch drop: Ten explanations for decreasing political trust in the Netherlands. International Review of Administrative Sciences.

[CR10] Bovens, M., & Wille, A. (2009). *Diploma democracy: On the tensions between meritocracy and democracy. Verkenning for the NWO Programma* ‘*Contested Democracies*’. Utrecht University/Leiden University

[CR11] Bovens M, Wille A (2010). The education gap in participation and its political consequences. Acta Politica.

[CR12] Bovens M, Wille A (2012). The education gap in participation: A rejoinder. Acta Politica.

[CR13] Braun M, Johnson TP, Harkness JA, Braun M, Edwards B, Johnson TP, Lyberg L, Mohler PP (2010). An illustrative review of techniques for detecting inequivalences. Survey methods in multinational, multiregional, and multicultural contexts.

[CR14] Brewer P, Aday S, Gross K (2005). Do Americans trust other nations? A panel study*. Social Science Quarterly.

[CR15] Campbell DT, Fiske DW (1959). Convergent and discriminant validation by the multitrait-multimethod matrix. Psychological Bulletin.

[CR16] Catterberg G, Moreno A (2006). The individual bases of political trust: Trends in new and established democracies. International Journal of Public Opinion Research.

[CR17] Cheung G, Rensvold R (2002). Evaluating goodness-of-fit indexes for testing measurement invariance. Structural Equation Modeling.

[CR18] Dalton RJ (2004). Democratic challenges, democratic choices: The erosion of political support in advanced industrialized democracies.

[CR19] Dalton RJ (2005). The social transformation of trust in government. International Review of Sociology.

[CR20] Davidov E, Coromina L (2013). Evaluating measurement invariance for social and political trust in Western Europe over four measurement time points (2002–2008). ASK. Research & Methods.

[CR21] Dekker, P. (2012). Political trust in the Netherlands. *Paper presented at 65th annual WAPOR conference*.

[CR22] Dekker P, van der Meer T, Schyns P, Steenvoorden E (2009). Crisis in aanocht: Verdiepingsstudie continu onderzoek burgerperspectieven 2008.

[CR23] Denters B, Gabriel O, Torcal M, Van Deth JW, Montero JR, Westholm A (2007). Political confidence in representative democracies Socio-cultural versus political explanations. Citizenship and involvement in European democracies: A Comparative analysis.

[CR24] Easton D (1965). A framework for political analysis.

[CR25] Easton D (1975). Reassessment of the concept of political support. British Journal of Political Science.

[CR26] Hakhverdian A, van der Brug W, de Vries C (2011). The emergence of a “diploma democracy”? The political education gap in the Netherlands, 1971–2010. Acta Politica.

[CR27] Hendriks F (2009). Contextualizing the Dutch drop in political trust: Connecting underlying factors. International Review of Administrative Sciences.

[CR28] Hetherington MJ, Rudolph TJ (2008). Priming, performance, and the dynamics of political trust. The Journal of Politics.

[CR29] Highton B (2009). Revisiting the relationship between educational attainment and political sophistication. The Journal of Politics.

[CR30] Hooghe M (2011). Why there is basically only one form of political trust. The British Journal of Politics & International Relations.

[CR31] Hooghe M, Marien S (2013). A comparative analysis of the relation between political trust and forms of political participation in Europe. European Societies.

[CR63] Jöreskog KG (1971). Simultaneous factor analysis in several populations. Psychometrika.

[CR32] Judd CM, Krosnick JA, Milburn MA (1981). Political involvement and attitude structure in the general public. American Sociological Review.

[CR33] Judd CM, Milburn MA (1980). The structure of attitude systems in the general public: Comparisons of a structural equation model. American Sociological Review.

[CR34] Klingemann H, Norris P (1999). Mapping political support in the 1990s: A global analysis. Critical citizens.

[CR35] Klingemann H, Fuchs D (1995). Citizens and the state.

[CR36] Kriesi H, Grande E, Lachat R, Dolezal M (2008). West European politics in the age of globalization.

[CR37] Levi M, Stoker L (2000). Political trust and trustworthiness. Annual Review of Political Science.

[CR38] Listhaug O, Wiberg M, Klingemann H, Fuchs D (1995). Confidence in political and private institutions. Citizens and the state.

[CR39] Little TD, Slegers DW, Card NA (2006). A non-arbitrary method of identifying and scaling latent variables in SEM and MACS models. Structural Equation Modeling.

[CR40] Luskin RC (1990). Explaining political sophistication. Political Behavior.

[CR41] Marien S, Hooghe M (2011). Does political trust matter? An empirical investigation into the relation between political trust and support for law compliance. European Journal of Political Research.

[CR42] Meuleman B, Davidov E, Billiet J (2009). Changing attitudes toward immigration in Europe, 2002–2007: A dynamic group conflict theory approach. Social Science Research.

[CR43] Newton K (2001). Trust, social capital, civil society, and democracy. International Political Science Review.

[CR44] Newton K, Zmerli S (2011). Three forms of trust and their association. European Political Science Review.

[CR45] Poznyak D, Meuleman B, Abts K, Bishop GF (2014). Trust in American government: Longitudinal measurement equivalence in the ANES, 1964–2008. Social Indicators Research.

[CR46] Quintelier E, Hooghe M (2011). Political attitudes and political participation: A panel study on socialization and self-selection effects among late adolescents. International Political Science Review.

[CR47] Rudolph T, Evans J (2005). Political trust, ideology, and public support for government spending. American Journal of Political Science.

[CR48] Saris WE, Gallhofer I (2007). Design, evaluation, and analysis of questionnaires for survey research.

[CR49] Saris, W. E., Satorra, A., & van der Veld, W. M. (2009). Testing structural equation models or detection of misspecifications? *Structural Equation Modeling: A Multidisciplinary Journal*. doi:10.1080/10705510903203433

[CR50] Savelkoul M, Scheepers P, van der Veld W, Hagendoorn L (2012). Comparing levels of anti-Muslim attitudes across Western countries. Quality & Quantity.

[CR51] Schnaudt C, Van Deth JW, Tausendpfund M (2013). Politisches Vertrauen. Politik im kontext: Ist alle politik lokale politik? Individuelle und kontextuelle determinanten politischer orientierungen.

[CR52] Steenbergen MR (2000). Item Similarity in Scale Analysis. Political Analysis.

[CR53] Steenkamp J, Baumgartner H (1998). Assessing measurement invariance in cross-national consumer research. Journal of Consumer Research.

[CR54] Thomassen JJ (2005). The European voter. A comparative study of modern democracies.

[CR55] Van der Brug W, van Praag P (2007). Erosion of political trust in the Netherlands: Structural or temporarily? A research note. Acta Politica.

[CR56] Van der Meer T (2010). In what we trust? A multi-level study into trust in parliament as an evaluation of state characteristics. International Review of Administrative Sciences.

[CR57] Van Deth JW (2000). Interesting but irrelevant: Social capital and the saliency of politics in Western Europe. European Journal of Political Research.

[CR58] Van Deth JW, Landman T, Robinson N (2009). Establishing equivalence. The sage handbook of comparative politics.

[CR59] Van Elsas E (2014). Political trust as a rational attitude: A comparison of the nature of political trust across different levels of education. Political Studies.

[CR60] Van Oorschot W, Meuleman B (2012). Welfarism and the multidimensionality of welfare state legitimacy: Evidence from The Netherlands, 2006. International Journal of Social Welfare.

[CR61] Zmerli, S. (2006). Political confidence in Europe. *Paper presented at international political science association 20th world congress*.

